# Tracing Emergence of SARS-CoV-2 Variants: Insights from Comprehensive Assessment Using Reverse Transcription Polymerase Chain Reaction and Whole Genome Sequencing

**DOI:** 10.3390/microorganisms13020311

**Published:** 2025-01-31

**Authors:** Duyeon Na, Yuna Hong, Chaeyeon Lee, Myungshin Kim

**Affiliations:** 1 Catholic Genetic Laboratory Center, Seoul St. Mary’s Hospital, College of Medicine, The Catholic University of Korea, Seoul 06591, Republic of Korea; msna09@catholic.ac.kr (D.N.); yuna1013@catholic.ac.kr (Y.H.); lcywin0813@catholic.ac.kr (C.L.); 2 Department of Medical Sciences, Graduate School of The Catholic University of Korea, Seoul 06591, Republic of Korea; 3 Department of Laboratory Medicine, College of Medicine, The Catholic University of Korea, Seoul 06591, Republic of Korea

**Keywords:** SARS-CoV-2, reverse-transcription polymerase chain reaction, whole-genome sequencing

## Abstract

The emergence and evolution of SARS-CoV-2 variants, such as Delta and Omicron, pose significant challenges to pandemic management. This study evaluated the effectiveness of reverse-transcription polymerase chain reaction (RT-PCR) and whole-genome sequencing (WGS) in detecting and characterizing SARS-CoV-2 variants using 624 samples collected in South Korea from mid-2021 to mid-2022. Two RT-PCR genotyping assays demonstrated a high concordance rate (90.4%) in identifying the Delta variant during its dominance. In contrast, WGS revealed extensive genetic diversity among Omicron sub-lineages, identifying 29 distinct sub-lineages, including two South Korea-specific variants (BA.1.1.5 and BA.2.3.8). Clustering analysis of WGS data highlighted distinct groupings of BA.1, BA.2, and BA.5 sub-lineages, with overlap in shared mutations suggesting evolutionary convergence. Sub-lineage diversity expanded during rapid transmission phases and subsequently consolidated as dominant lineages emerged. These findings highlight the complementary strengths of RT-PCR and WGS and underscore the importance of integrating these methodologies for effective variant monitoring and public health response.

## 1. Introduction

The COVID-19 pandemic caused by the novel coronavirus SARS-CoV-2 continues to pose significant challenges to global health systems [1,2,3]. The emergence of highly transmissible and potentially more severe variants, such as the Delta and Omicron variants, has further complicated the situation [4,5]. The Delta variant, first identified in late 2020, rapidly became a dominant strain worldwide [6]. It is known for its increased transmissibility and association with higher rates of hospitalization and mortality [7]. The Omicron variant, identified in late 2021, raised additional concerns due to its numerous mutations and potential immune escape capabilities [8,9].

Understanding the dynamics of these variants and developing effective strategies to respond at a local center level are crucial. Monitoring the prevalence and impact of these variants within a local center context is essential for guiding local responses and implementing effective containment measures.

Diagnostic nucleic acid amplification testing techniques, such as RT-PCR, have become reliable tests for detecting viruses like SARS-CoV-2. These techniques offer rapid detection and high sensitivity, enabling the diagnosis of infections even in the early stages. Despite its advantages, nucleic acid amplification is associated with the risk of false-negative or false-positive results [10]. The rapid emergence of SARS-CoV-2 variants is a key factor that can reduce diagnostic testing sensitivity. SARS-CoV-2 exhibits a high mutation rate, and certain variants can affect diagnostic target regions, reducing the sensitivity and specificity of diagnostic nucleic acid amplification testing [11]. Therefore, genotyping is an essential method for tracking and identifying SARS-CoV-2 variants, enabling more accurate virus detection and improving diagnostic sensitivity. Genotyping makes it possible to achieve sensitive diagnosis of specific SARS-CoV-2 variants, thereby enabling disease prediction. Genotyping can identify new pathogen variants and accurately detect specific mutations. This approach can enhance the sensitivity of diagnostic nucleic acid amplification tests, overcome their limitations, and reduce the occurrence of false-negative and false-positive results. However, it has limitations, including high costs, the need for advanced technical expertise, and challenges in rapidly analyzing newly evolving variants. Furthermore, it requires longer analysis times than diagnostic nucleic acid amplification tests [12]. As mentioned earlier, genotyping poses challenges in diagnosing SARS-CoV-2 because of its high cost and the need for advanced technical expertise. To overcome these limitations, combining genotyping with other diagnostic nucleic acid amplification techniques, such as RT-PCR, can be proposed as a solution. This approach allows more accurate variant detection, enhances sensitivity, and mitigates the limitations inherent to each individual technique.

In this paper, we share real-world experiences of detecting Delta and Omicron variants of SARS-CoV-2 by comparing two prevalent variant detection techniques: reverse-transcription polymerase chain reaction (RT-PCR) and whole-genome sequencing (WGS) using next-generation sequencing. RT-PCR stands out for its targeted detection of mutations specific to Delta and Omicron variants, offering a swift and economical solution for variant screening [13]. In contrast, WGS provides an expansive view of the viral genome, facilitating the identification of emerging mutations and enabling a richer profiling of sub-lineages [14]. By juxtaposing these techniques, our goal was to pinpoint the most effective strategy for timely and precise detection of evolving SARS-CoV-2 variants.

We aimed to investigate the evolving patterns of SARS-CoV-2 Delta and Omicron variants within a specific institutional setting, comparing these dynamics with global trends. This study utilized RT-PCR and WGS to track variant evolution and prevalence, providing critical insights into the detection and monitoring of SARS-CoV-2 variants in real-world clinical settings.

## 2. Materials and Methods

### 2.1. Study Design and Samples

This study was approved by the Institutional Review Board (IRB) of the Catholic Medical Center, Seoul, Republic of Korea (XC21TIDI0118). The IRB waived the requirement for informed consent due to the retrospective nature of the study. Between June 2021 and August 2022, we conducted a comprehensive analysis of 624 clinical samples from subjects who were diagnosed with COVID-19, collected from two university hospitals in Seoul and Incheon, South Korea. Samples were divided into four periods based on the techniques used to identify variants: Period A (1 June 2021–22 September 2021), Period B (1 December 2021–10 January 2022), Period C (11 January 2022–8 February 2022), and Period D (20 June 2022–12 August 2022).

Most patients visited the COVID-19 clinic for virus screening using RT-PCR; therefore, information on symptoms and severity was not obtained. Among the 114 patients for whom clinical severity could be assessed through medical record review, eight showed a severity score of 2 or greater [15]. Six of these patients were treated for hematologic malignancies, two had chronic obstructive pulmonary disease, and one was of post-operative status following distal common bile duct cancer. These included three cases of BA.2.3, two of BA.2.3.8, three of BA.5.2, and one of BA.5.2.1.

**Figure 1:**
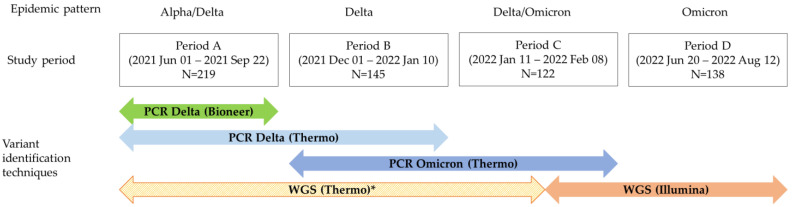
Timeline of genotyping strategies and assay utilization for SARS-CoV-2 variant identification across four periods. WGS, whole-genome sequencing. * WGS was selectively performed on samples with discrepancies or unidentified variants.

The genotyping strategy was modified over time (Figure 1). During Period A, we used the TaqMan Delta assay for SARS-CoV-2 variant typing (Thermo Fisher Scientific, Waltham, MA, USA) and AccuPower RT-PCR (Bioneer Corporation, Daejeon, Republic of Korea) assays for all 219 samples. In Period B, we utilized the TaqMan Delta and TaqMan Omicron assays (Thermo Fisher Scientific, USA) for variant typing of 145 samples. In Period C, only the TaqMan Omicron assay was used for variant typing for 122 samples. For mutation analysis, the Ion AmpliSeq SARS-CoV-2 research panel (Thermo Fisher Scientific, USA) was used during Periods A and B and early Period C, but only for cases with discrepancies between assays or unidentified variants. In later Period C, the Illumina COVIDSeq assay (Illumina Inc., San Diego, CA, USA) was introduced. During Period D, dominated by the Omicron variant, we performed WGS using the Illumina COVIDSeq Assay to investigate Omicron sub-lineage patterns in 138 samples.

### 2.2. SARS-CoV-2 Variant Detection by RT-PCR

The AccuPower^®^ SARS-CoV-2 variant ID real-time RT-PCR kit targeted the Delta variant (L452R, E484Q, P681R). RNA from each sample was added to a reaction mix, followed by reverse transcription and amplification. A CFX 96 real-time thermal cycler was used to analyze Ct values and fluorescence intensities. RT-PCR was performed using TaqMan COVID-19 Delta and Omicron assays targeting specific SARS-CoV-2 variants, including L452R, E484Q, and P681R for the Delta variant, and G339D and Q493R for the Omicron variant. These assays were conducted using the TaqMan™ Fast Virus 1-Step Master Mix and subsequently analyzed using QuantStudio™Design & Analysis software (version 1.5.2).

### 2.3. SARS-CoV-2 Variant Detection by WGS

The Ion AmpliSeq™ SARS-CoV-2 research panel, developed by Thermo Fisher Scientific, was designed exclusively for the Ion Torrent sequencing system. Covering over 99% of the SARS-CoV-2 genome, including crucial open reading frames and untranslated regions, this panel enables comprehensive genetic analysis. Using multiplex PCR and the Ion AmpliSeq Library Kit 2.0, 10 ng of total RNA was subjected to PCR amplification and amplicons were ligated with Ion Xpress Barcode Adapters. An Ion Library TaqMan quantitation kit was used to ensure quality. Templating on Ion Sphere Particles, enrichment via the Ion OneTouch ES system, and sequencing on the Ion GeneStudio S5 system were then performed. Sequence analysis was performed using Torrent Suite v5.12. Variant calling was performed with a Torrent Variant Caller plugin supplemented by Ion Reporter software available online: https://ionreporter.thermofisher.com (accessed on 24 February 2024), for annotation and interpretation to provide detailed insights into SARS-CoV-2 genetic characteristics. Leveraging the Ion AmpliSeq™ SARS-CoV-2 research panel, this approach allowed for nuanced exploration of the virus’s genetic makeup.

The Illumina COVIDSeq Assay, purpose-built for detecting and analyzing SARS-CoV-2 variants, involved a meticulous workflow. Starting with cDNA synthesis from RNA samples, a multiplex PCR protocol was used to amplify specific viral genome regions. PCR-amplified product then underwent tagmentation and adapter ligation for subsequent sequencing on a MiSeq platform, known for its precision. Data analysis utilized the advanced DRAGEN COVIDSeq Test Pipeline with ANNOVAR to facilitate variant annotation. To enhance validity, data were integrated from the Global Initiative on Sharing All Influenza Data (GISAID) database. Resulting genome sequences were aligned and phylogenetically analyzed, masking problematic variant positions. Lineage classification employed Phylogenetic Assignment of Named Global Outbreak Lineages (PANGOLIN) software (version 4.3.1). By integrating Illumina COVIDSeq assays, we unraveled intricate Omicron variant patterns, enriching our understanding of viral evolution and informing pandemic response strategies.

### 2.4. Phylogenetic Tree Based on Mutational Profiles of SARS-CoV-2 Lineages

Hierarchical cluster analysis of the Omicron variant was conducted using SPSS version 24.0. The between-groups linkage method was employed, with squared Euclidean distance serving as the similarity measure. Prior to clustering, the variables were standardized to Z scores to ensure equal weighting. The resulting dendrogram was analyzed to determine the optimal number of clusters, facilitating the identification of groups that shared similar characteristics within the dataset.

## 3. Results

### 3.1. Similarities in Regional and Global COVID-19 Subtype Patterns over the Study Period

**Figure 2:**
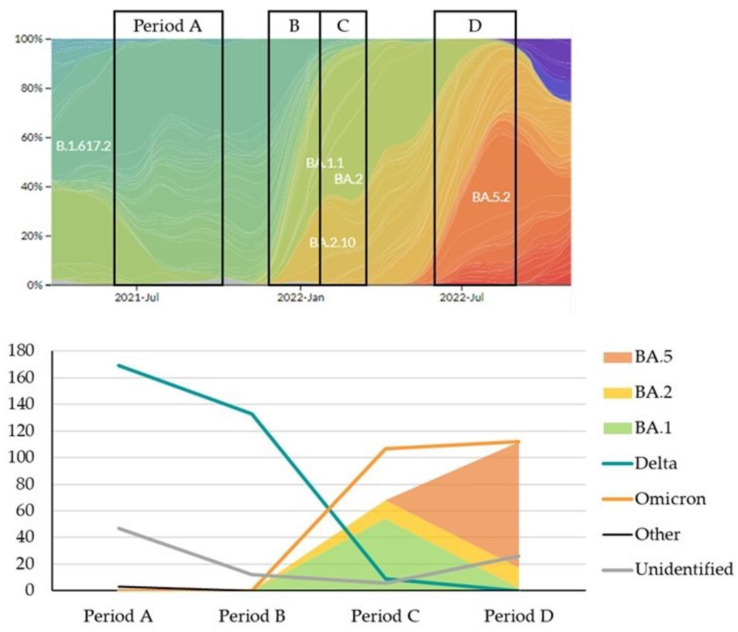
Temporal distribution and prevalence of SARS-CoV-2 strains across defined study periods. The upper panel, adapted from Nextstrain filtered to Asia, illustrates the changing prevalence of SARS-CoV-2 strains, with each period (A, B, C, D) marked by vertical black lines. The colored regions represent the dominant strains during each period, with gradual transitions indicating shifts in prevalence. The lower panel presents the absolute count of identified strains within each period. This panel highlights the emergence and decline of different strains over time.

Figure 2 illustrates the changes in the prevalence of SARS-CoV-2 variants in Nextstrain focused on Asia alongside our data. The genotyping results across different time periods (A, B, C, and D) demonstrate a dynamic shift in SARS-CoV-2 variants over time, as seen in Asia (upper panel). In Period A, the B.1.617.2 (Delta) variant was predominant. As we transitioned into Period B, there was a notable emergence of the BA.1 and BA.2 Omicron sub-lineages, with BA.2 becoming more prevalent by the end of this period. During Period C, BA.2 continued to dominate, gradually being replaced by the BA.2.10 sub-lineage. By Period D, BA.5.2 had become the dominant sub-lineage, nearly entirely replacing other variants, as BA.5 emerged as the primary Omicron sub-lineage.

The number of cases associated with each variant or sub-lineage in our cohort closely resembled those observed across Asia. Delta had the highest count during Period A, but drastically decreased in Period B as Omicron variants emerged. The BA.2 sub-lineage surged in Period B and remained prominent through Period C. By Period D, BA.5 had shown a sharp increase, dominating the variant distribution. This progression reflects the SARS-CoV-2 evolution trajectory in Asia, with our cohort experiencing similar variant shifts.

### 3.2. Concordance and Validation of TaqMan and AccuPower RT-PCR Assays for COVID-19 Delta Variant Detection

Table 1 shows comparative findings between TaqMan and AccuPower RT-PCR assays regarding the detection of the SARS-CoV-2 Delta variant during the Delta-dominant period (Period A). Out of 219 samples, a significant concordance rate of 90.4% (198/219) was observed between the TaqMan and AccuPower assays. The TaqMan assay identified 166 samples as Delta variant-positive, with 153 confirmed by the AccuPower assay. Of the 53 samples not identified as Delta by TaqMan, 49 were also classified as non-Delta (other variant) by AccuPower. We subjected the 21 discordant results to WGS (Ion AmpliSeq) testing, but only 10 samples yielded results, possibly due to the low viral load. Of these samples, WGS testing confirmed that eight were Delta (B.1.617.2). The remaining two samples were identified as non-Delta, specifically B.1.619.1 and B.1.620, which were prevalent in South Korea during the early stages of the outbreak [16]. These findings suggest that the TaqMan assay had better sensitivity for detecting the Delta variant compared to the AccuPower assay.

### 3.3. Genomic Sub-Lineage and Mutation Distribution of the Omicron Variant

A total of 208 Omicron samples were subjected to genomic lineage analysis using WGS. However, 27 samples were not available for sub-lineage analysis. During Period C, BA.1 was predominant, accounting for 72.2% (52/72 samples). BA.2, initially comprising a relatively small proportion, started to increase in Period D. An additional finding observed in Period D was the emergence of BA.5, which became predominant in the latter part of Period D.

**Figure 3:**
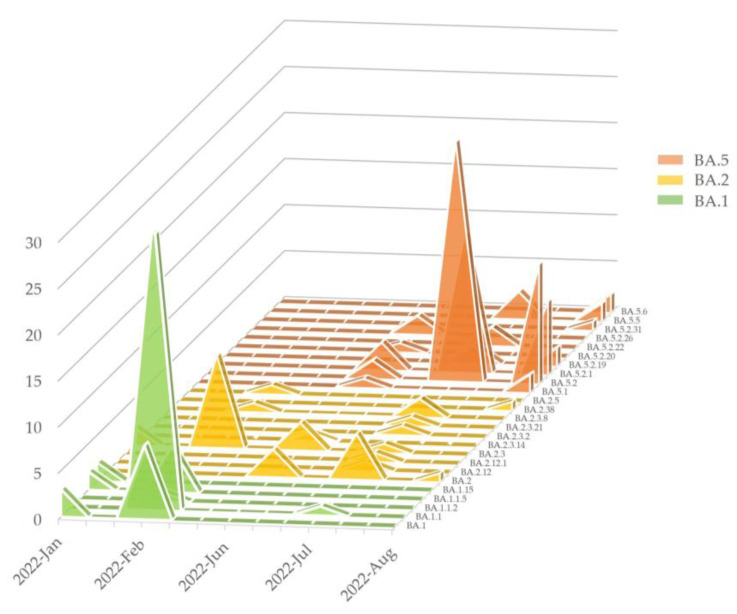
Genomic sub-lineage analysis of the Omicron variant. Detailed analysis of sub-lineages according to time period.

We identified 29 unique sub-lineages of Omicron, illustrating the immense genetic diversity and adaptability of the variant. Among BA.1, five sub-lineages were identified (BA.1, BA.1.1, BA.1.1.2, BA.1.1.5, and BA.1.15), with BA.1.1 being predominant (61.1%, 33/54). Specifically, the BA.1.1.5 sub-lineage was found in seven samples and was recognized as a South Korea-specific lineage (alias of B.1.1.529.1.1.5). Among BA.2, 10 sub-lineages were identified (BA.2, BA.2.12, BA.2.12.1, BA.2.3, BA.2.3.14, BA2.3.2, BA.2.3.21, BA.2.3.8, BA.2.38, and BA.2.5), with BA.2.3 being predominant (47.1%, 16/34) followed by BA.2 (23.5%, 8/34). The number of identified sub-lineages started with two, increased to seven in July 2022, and then decreased. The sub-lineage BA.2.3.8 was detected in three samples and reported in seven countries, including South Korea, during January to August 2022. Among BA.5, 10 sub-lineages were identified (BA.5.1, BA.5.2, BA.5.2.1, BA.5.2.19, BA.5.2.20, BA.5.2.22, BA.5.2.26, BA.5.2.31, BA.5.5, and BA.5.6), with BA5.2 being predominant (47.7%, 41/86) followed by BA.5.2.1 (29.1%, 25/86) (Figure 3).

**Figure 4:**
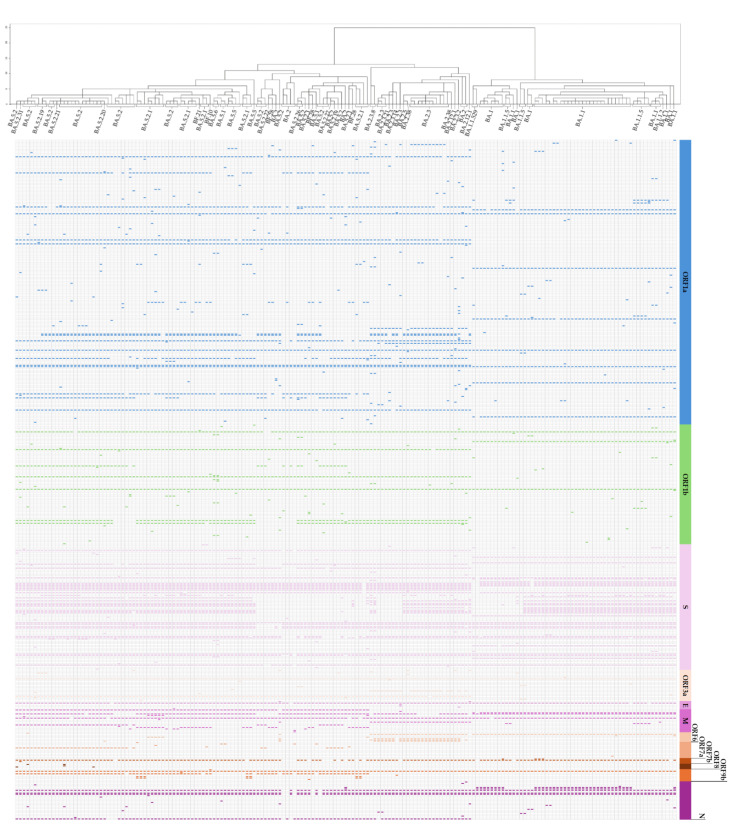
Genomic sub-lineage and mutation distribution of the Omicron variant. Mutation distribution across genes in the Omicron variant detected using whole-genome sequencing. Each column represents a specific gene, and each dot indicates the presence of a mutation in a specific sub-lineage. The hierarchical clustering on the left displays the phylogenetic relationships among 29 Omicron sub-lineages.

Mutations were found across genes (Figure 4), including ORF1a, ORF1b, S, ORF3a, E, M, ORF6, ORF7a, ORF7b, ORF8, ORF9b, and N. The S gene, associated with the spike protein, showed particularly extensive mutation prevalence across multiple sub-lineages. A total of 29 unique Omicron sub-lineages were identified, highlighting the remarkable genetic diversity and adaptability of the Omicron variant. Specifically, the BA.1.1.5 lineage was found in seven samples. It was recognized as a South Korea-specific lineage (Alias of B.1.1.529.1.1.5). The hierarchical dendrogram representation provided a clear picture of lineage evolution, highlighting points of genetic convergence and divergence.

Clustering analysis revealed that BA.1 formed a distinct group, separated from BA.2 and BA.5. While BA.2 and BA.5 were largely segregated into separate clusters, some overlap was observed due to shared genetic mutations, indicating areas of convergence in their evolution during the transition from BA.2 to BA.5.

## 4. Discussion

The ongoing evolution of the SARS-CoV-2 virus, exemplified by the Delta and Omicron variants, underscores the critical need for robust variant-monitoring strategies. This study captured the dynamic shift in variant dominance, from Delta to Omicron, within a South Korean institutional setting from mid-2021 to mid-2022. Through a multi-pronged approach combining RT-PCR and WGS, we not only tracked these transitions but also unveiled critical insights into the genetic diversity and adaptability of the Omicron variant [17,18].

RT-PCR and WGS both have distinct advantages and limitations for detecting SARS-CoV-2 variants [19,20,21]. RT-PCR offers rapid and cost effective, mutation-specific detection, but is limited in granularity [22,23]. In contrast, WGS, while resource-intensive, provides comprehensive genomic insights essential for tracking viral evolution [24,25,26]. RT-PCR demonstrated high concordance (90.4%) in detecting the Delta variant, underscoring its value as a rapid, cost-effective diagnostic tool during early variant surges. However, as the Omicron variant emerged, WGS proved indispensable for capturing its extensive genetic diversity, identifying 29 unique sub-lineages, including two South Korea-specific lineages (BA.1.1.5 and BA.2.3.8) [27,28].

Our results also reveal the complementary strengths and limitations of the diagnostic techniques. While RT-PCR assays provided a swift response during the Delta-dominant period, the discrepancies highlight the need for supplementary methods like WGS. The nuanced exploration offered by WGS, including detailed mutational analyses, enabled us to map the Omicron variant’s evolutionary trajectory and identify regional sub-lineages.

One of the most striking findings is the observed increase and subsequent decline in sub-lineage diversity with the spread of each lineage, particularly Omicron. This pattern suggests a potential adaptive saturation, where genetic diversity expands during rapid transmission phases and consolidates as dominant sub-lineages outcompete others [29]. These insights could guide predictive models of variant evolution, offering a framework for anticipating the emergence of future variants.

Genotyping can provide crucial information for predicting the likelihood of disease manifestation. In the SARS-CoV-2 Omicron variant, gene expression varies according to sub-lineage type. The S protein’s receptor-binding domain (RBD) binds to the human angiotensin-converting enzyme 2 (hACE2) receptor, allowing the SARS-CoV-2 virus to enter our bodies [30]. BA.1, the first identified and most common sub-lineage of the Omicron variant, harbors unique mutations, such as G446S and G496S. BA.2 harbors unique mutations in the S protein RBD, namely, R408S and S317F, which enhance the binding affinity between the RBD and the hACE2 receptor, contributing to its high transmissibility [31]. BA.5 harbors the F486V mutation in the RBD, which is considered a major factor contributing to its infectivity. Additionally, the N679K and P681H mutations induced amino acid changes near the furin cleavage site, promoting the cleavage of the S1 and S2 subunits of the spike protein. This enhances the binding affinity to the hACE2 receptor, thereby increasing the infectivity of the virus. Understanding the mutations of evolving sub-lineages and their impact on hACE2 receptor binding can help predict the pathogenicity of emerging variants, enabling the development of preventive therapies and proactive response strategies [29,32].

Genotyping can significantly contribute to the development of vaccines targeting specific mutations. Nonsynonymous mutations, which alter the amino acid sequence and structure of the S protein, enhance the virus’s ability to replicate, thereby increasing its transmissibility. This finding highlights the importance of considering the S gene in vaccine development because it can enhance transmissibility and immune evasion, potentially reducing vaccine efficacy [33,34]. In particular, the Omicron variant contains a greater number of nonsynonymous mutations than other variants, which play a key role in enhancing immune escape capabilities. Mutations such as Q498R and E484A in the RBD and NTD of the S protein alter the antigenicity and disrupt the binding of neutralizing antibodies. Additionally, the Omicron variant’s NTD contains three small deletions, four substitutions, and a three-residue insertion, which further facilitate immune escape [35,36]. In our study, BA.2 and BA.5 were clustered separately, but overlapping genetic mutations demonstrated convergent evolution. Among them, the shared mutations F846V and D405N in BA.2 and BA.5 alter the structure of the RBD, hindering the binding of neutralizing antibodies and playing a critical role in immune escape [37]. This suggests that targeting shared mutations across lineages in vaccine design can enhance the binding efficacy of neutralizing antibodies and strengthen responses against evolving SARS-CoV-2 lineages.

Using shared variants observed among evolving variants can aid in developing effective vaccine design strategies. A relevant example is the bivalent booster formulations of the Moderna and Pfizer–BioNTech vaccines, which were designed based on the mRNA of the ancestral SARS-CoV-2 virus and certain mutations from the Omicron lineage, including BA.5. Clinical results demonstrate that the SARS-CoV-2 bivalent booster provides a 33.5-percentage-point-higher preventive efficacy in reducing severe infections compared to the monovalent booster, highlighting its improved effectiveness [38,39]. Furthermore, analyzing the sub-lineages of variants circulating across different regions and time periods can provide critical insights for designing vaccines capable of addressing multiple lineages. COVID-19 Virus-Human Outcomes Prediction (ViHOP) and similar personalized clinical risk assessment tools can be utilized to predict clinical outcomes and advance vaccine design. Utilizing clinical databases such as ViHOP to update our sub-lineage analysis could serve as a crucial method for responding in real time to the impacts of emerging SARS-CoV-2 variants [40]. Genotyping makes it possible to understand mutation characteristics and integrate mutation-based analyses with data, enabling the development of effective vaccines. Furthermore, it will enable the establishment of real-time response strategies to address emerging variants.

The extensive mutations observed across multiple genes, particularly in the S gene associated with the spike protein, underscore the genetic complexity and adaptability of the Omicron variant [41]. The hierarchical dendrogram and clustering analyses further illustrate the evolutionary relationships among Omicron sub-lineages. The distinct grouping of BA.1 and the relative segregation of BA.2 and BA.5 into separate clusters provide a detailed view of Omicron’s diversification. Interestingly, the observed overlap in genetic mutations between BA.2 and BA.5 suggests potential areas of convergence in their evolutionary paths, likely driven by selective pressures favoring shared advantageous traits. This convergence during the transition from BA.2 to BA.5 reflects the dynamic interplay between mutation and selection, underscoring the adaptive flexibility of SARS-CoV-2.

Therefore, the genotyping data from our study can be used to predict the expression of specific mutations and the likelihood of early-stage disease. In this study, the observation of overlapping genetic mutations in BA.2 and BA.5 sub-lineages within clusters suggests a potential association with characteristics such as immune evasion and increased transmissibility. Future studies are necessary to determine whether these mutations contribute to the increased risk of disease manifestation. As a result, our research has the potential to predict the likelihood of disease manifestation and lay the foundation for developing countermeasures against severe diseases and personalized treatments for patients. Furthermore, genotyping results could serve as essential foundational data for such disease prediction and treatment development.

However, our study has limitations. Firstly, the timeframe, spanning four periods from mid-2021 to mid-2022, may not fully capture the complete evolutionary trajectory of variants. Secondly, although the samples were collected from two hospitals in different areas, the findings may not be generalizable to the broader population or other global settings. Lastly, not all samples underwent WGS, limiting the depth of sub-lineage analysis. The limitations of our study can offer valuable insights for shaping future research efforts. Since we have only analyzed four periods, a more complete evolutionary trajectory of the variant can be captured by extending the analysis to subsequent periods. As our study samples were collected from two different regions and two hospitals, expanding the sample collection to include diverse regions or population groups could provide a basis for generalization and help identify variations specific to certain groups. Finally, combining RT-PCR and WGS techniques to develop highly sensitive and cost-effective approaches could augment the depth of detailed sub-lineage analysis.

In conclusion, this study highlights the value of employing diverse testing methodologies for comprehensive and accurate detection of COVID-19 variants. Our findings emphasize the dynamic nature of Omicron’s evolution and the importance of genomic analyses in guiding public health strategies. Each lineage of SARS-CoV-2 variants possesses unique mutations, and the detected mutations influence transmissibility, immune escape, and the disruption of neutralizing antibody binding. Furthermore, the analysis of SARS-CoV-2 variants provides crucial insights for targeting the infectivity and transmissibility of evolving sub-lineage mutations or designing vaccines capable of responding to multiple lineages.

## Figures and Tables

**Table 1:** Comparison of TaqMan and AccuPower RT-PCR assay results for SARS-CoV-2 Delta variants during Period A.

	TaqMan	Delta	Non-Delta	Total
AccuPower				
Delta		149 (68.0)	4 (1.8)	153
Non-Delta		17 * (7.8)	49 (22.4)	66
Total		156	53	219

Data are expressed as number of cases (%). * Ten of the cases were confirmed by whole-genome sequencing as B.1.617.2 (Delta) (*n* = 8), B.1.619.1 (*n* = 1), and B.1.620 (*n* = 1). Abbreviation: RT-PCR, reverse-transcription polymerase chain reaction.

## Data Availability

The raw data supporting the conclusions of this article will be made available by the authors on request due to ethical restrictions and privacy concerns.
